# New race-free creatinine- and cystatin C-based equations for the estimation of glomerular filtration rate and association with cardiovascular mortality in the Athero*Gene* study

**DOI:** 10.1007/s11739-023-03529-9

**Published:** 2024-02-13

**Authors:** George Ntaios, Jan Brederecke, Francisco M. Ojeda, Tanja Zeller, Stefan Blankenberg, Renate B. Schnabel

**Affiliations:** 1 Department of Internal Medicine, School of Health Sciences, University of Thessaly, 41110 Larissa, Greece; 2 Department of Cardiology, University Center of Cardiovascular Science, University Heart and Vascular Center Hamburg-Eppendorf, University Medical Center Hamburg-Eppendorf, Hamburg, Germany; 3 German Center for Cardiovascular Research (DZHK) Partner Site, Hamburg, Germany

**Keywords:** Estimated glomerular filtration rate, Estimation of renal function, Mortality, Cardiovascular death, Prediction

## Abstract

Renal function is associated with cardiovascular outcomes and mortality. Among equations used to eGFR, CKD–EPI equations show more accurate association with cardiovascular risk and mortality than MDRD. Recently, new CKD–EPI equations were proposed which do not include race and would be considered sufficiently accurate to estimate eGFR in clinical practice. It is unknown if these new race-free equations are comparably well associated with cardiovascular outcomes in high-risk individuals. The analysis was performed in the Athero*Gene* Study cohort including patients at high cardiovascular risk. eGFR was determined using the established as well as the recently developed formulas which are calculated without the otherwise existing coefficient for black race. The outcome was cardiovascular death. Analyses included Cox-proportional hazard regression and area-under-the-curve calculation. The analysis included 2089 patients followed up for a median of 3.8 years with a maximum of 6.9 years, corresponding to an overall period of 7701 patient-years. Cardiovascular death occurred in 93 (4.45%), corresponding to an annualized rate of 1.2/100 person-years. In all Cox regression analyses, the estimated adjusted GFR was an independent predictor of cardiovascular death. The equations which included cystatin C showed higher C-index compared to those which did not include cystatin C (0.75–0.76 vs. 0.71, respectively). The equations for the estimation of eGFR which include cystatin C are better associated with cardiovascular death compared to the race-free equations which include only creatinine. This finding adds on the related literature which supports the elimination of race in GFR-estimating equations, and promotion of the use of cystatin C.

**Supplementary Information:**

The online version contains supplementary material available at 10.1007/s11739-023-03529-9.

## Introduction

The assessment of renal function is an integral component of daily clinical practice. It is typically determined by the estimated glomerular filtration rate (eGFR) which can be calculated with several proposed equations which take into consideration endogenous filtration markers, such as creatinine or cystatin C. The Modification of Diet in Renal Disease (MDRD) study equation was proposed in 1999 and uses four variables: sex, age, serum creatinine, and race [1]. The Chronic Kidney Disease Epidemiology Collaboration (CKD–EPI) creatinine-based equation, proposed in 2009, consists of the same four covariates and appears to provide a more accurate estimate of renal function than MDRD [2]. In 2012, the CKD–EPI combined creatinine–cystatin C equation was proposed and shown to perform better than equations based on either of these markers [3]. In 2021, the American Society of Nephrology and the National Kidney Foundation recommended that race should not be included in equations used to estimate renal function, as the use of race in clinical algorithms normalizes and reinforces misconceptions of racial determinants of health and disease [4]. Accordingly, the CKD–EPI collaboration recently proposed new equations which do not include race and would be considered sufficiently accurate to estimate eGFR in clinical practice [5]. Among them, the equations based on both serum creatinine and cystatin C estimated GFR more accurately than equations with either creatinine or cystatin C alone [5]. However, equations based on cystatin C are not yet widely adopted mainly because cystatin C measurement has not been broadly implemented across health systems or laboratories, partly due to its higher cost compared to creatinine.

Initially, the role of eGFR equations in clinical practice was to detect the onset of renal failure, to adjust the dose of drugs excreted by the kidneys, and to assess the effectiveness of strategies aiming to delay the progression of renal disease [1]. Soon, it became apparent that renal function is an important predictor of cardiovascular events and cardiovascular and all-cause mortality [6]. Accordingly, the 2019 European Society of Cardiology Guidelines for the management of dyslipidemias classify patients with moderate and severe CKD as of high and very high cardiovascular (CV) risk, respectively [7]. Among eGFR equations, the CKD–EPI equations showed a more accurate association with cardiovascular risk than the MDRD equation. However, it is not known if the new race-free CKD–EPI equations are accurately associated with cardiovascular events and mortality. In this context, our study assessed the association between the newly proposed race-free CKD–EPI equations with cardiovascular mortality.

## Methods

The analysis was performed in the Athero*Gene* cohort, which consists of patients that presented coronary artery disease undergoing coronary angiography at the Department of Cardiology at the University Medical Center of the Johannes Gutenberg-University in Mainz, Germany and the Department of Internal Medicine of the Federal Armed Forces Central Hospital in Koblenz, Germany, between 1996 and 2004. Of the screened patients, those with at least one stenosis of ≥ 30% angiographic lumen reduction in one major coronary artery were included. Of the 3795 enrolled participants, the present study uses those with complete information in the variables analyzed (*n* = 2089). The Athero*Gene* study has been described in detail before [8, 9].

### Risk factor assessment

Cardiovascular risk factor assessment in the Athero*Gene* study comprised the following variables: a mean blood pressure of 140 mm Hg (systolic) over 90 mm Hg (diastolic) was defined as arterial hypertension and participants taking antihypertensives were also classified as having arterial hypertension regardless of their actual blood pressure. The smoking status was coded as ever smoking or never smoking (cessation > 40 years or no smoking at all) and diabetes mellitus was defined by oral blood glucose lowering therapy and/or substitution of insulin. Participants with a diagnosis of dyslipidemia from a general practitioner or a low-density lipoprotein (LDL)/high-density (HDL) cholesterol ratio above 3.5 were classified as having dyslipidemia. Blood biomarkers were measured by routine methods. Cystatin C was determined from plasma samples by immunonephelometry using a Behring Nephelometer II (Dade-Behring, Inc.) [9, 10].

Estimated glomerular filtration rate was calculated using the race-based [2] as well as the recently developed race-free equations [3, 5] (Supplemental table). In particular, we assessed the original creatinine-only based CKD–EPI equation omitting the black race coefficient (termed *2009 CKD–EPI creatinine; eGFRcr(ASR-NB), new*); the original creatinine-only based CKD–EPI equation including the black race coefficient (termed 2009 CKD–EPI creatinine; eGFRcr(ASR), current), the revised race-free creatinine-only based CKD–EPI equation (termed 2021 CKD–EPI creatinine; eGFRcr(AS), new); the race-free cystatin C only based CKD–EPI equation (*termed 2012 eGFRcys[AS]*); the original equation including both serum creatinine and cystatin C and including the black race coefficient (termed *2012 CKD–EPI creatinine–cystatin C; eGFRcr-cys(ASR), current*), the original equation including both serum creatinine and cystatin C omitting the black race coefficient (termed *2012 CKD–EPI creatinine–cystatin C; eGFRcr-cys(ASR-NB), new*) and the race-free equation including both creatinine and cystatin C (termed *2021 eGFRcr-cys[AS], new*).

Our study cohort consisted only of Caucasians. For this reason, equations for *2009 CKD–EPI creatinine; eGFRcr(ASR), current* and *2009 CKD–EPI creatinine; eGFRcr(ASR-NB), new* will produce identical results as the only difference is the use of the multiplicative factor that is used for black individuals. The same applies to Eqs. *2012 CKD–EPI creatinine–cystatin C; eGFRcr-cys(ASR), current* and *2012 CKD–EPI creatinine–cystatin C; eGFRcr-cys(ASR-NB), new*.

### Follow-up period and outcomes’ definition

The median follow-up duration was 3.7 (3.8) years with a maximum of 6.9 years. Follow-up data were collected via postal questionnaires or telephone interviews by medical technicians. Three cardiologists conducted the validation of outcome events based on written medical reports obtained from general practitioners as well as hospital records. The outcome was cardiovascular death.

### Statistical analyses

Categorical variables were described by their absolute and relative frequencies and continuous variables by their quartiles. Cox regression analyses were performed for each eGFR equation for the outcome. Cox regression models were adjusted for available cardiovascular risk factors arterial hypertension, diabetes, smoking, dyslipidemia, and body mass index. Crude incidence rates of the outcome were calculated using Poisson regressions. Additionally, receiver operating characteristics (ROC) curves and the area under the curve (AUC) were calculated for cardiovascular mortality after 4 years for all GFR-estimating equations using the previously described methods [11]. Moreover, adjusted 1 year CVD mortality probability was calculated using Cox-proportional hazard models per eGFR formula for an example white woman with the lowest risk category for categorical covariates (smoking status, diabetes status, and BP category) and the overall mean values of available continuous covariates (age, body mass index, LDL and HDL cholesterol, log triglycerides, and log CRP). All calculations were done using R version 4.0.5 [12].

## Results

**Fig. 1:**
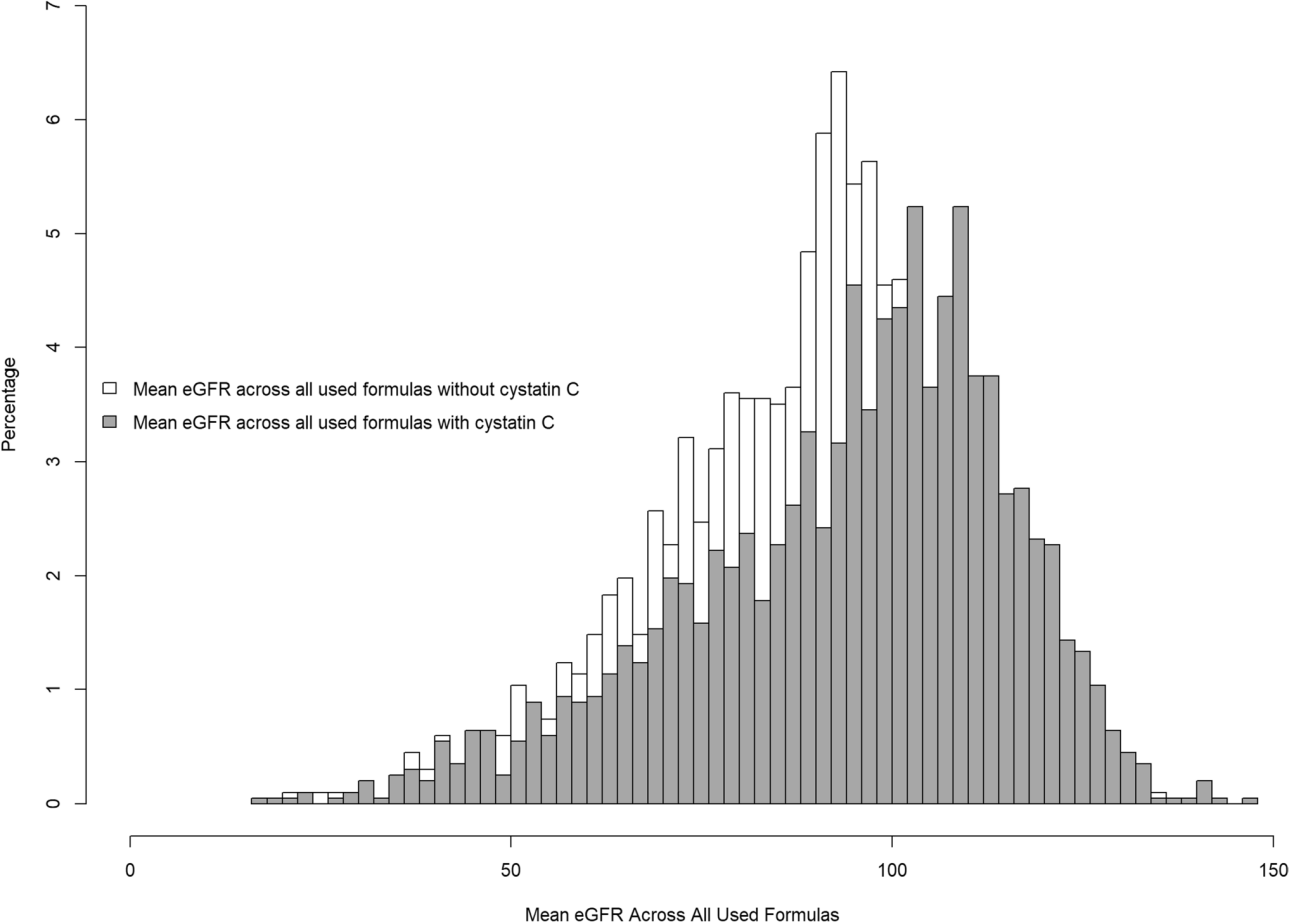
Histogram of the mean estimated glomerular filtration rate of all used equations

Among the initial cohort of 3800 patients, we included 2089 patients who had complete data about age, sex, cystatin C, creatinine, time to cardiovascular death, cardiovascular death, BMI, diabetes mellitus, current smoking, dyslipidemia, and hypertension. In patients excluded because of incomplete data, cystatin C was the main missing parameter (1563 patients). The median age was 63.0 years, and 77.6% were men. The median creatinine and cystatin C levels were 0.9 mg/dl and 0.8 mg/dl, respectively. The distribution of eGFR values among patients is presented in Fig. 1. The baseline characteristics of the patients are summarized in the supplemental table.

Patients were followed up for a maximum of 6.9 years and a median of 3.8 years, corresponding to an overall follow-up period of 7701 patient-years. During this period, the outcome of the outcome of cardiovascular death occurred in 93 (4.45%), corresponding to an annualized rate of 1.2 per 100 person-years, respectively.

**Fig. 2:**
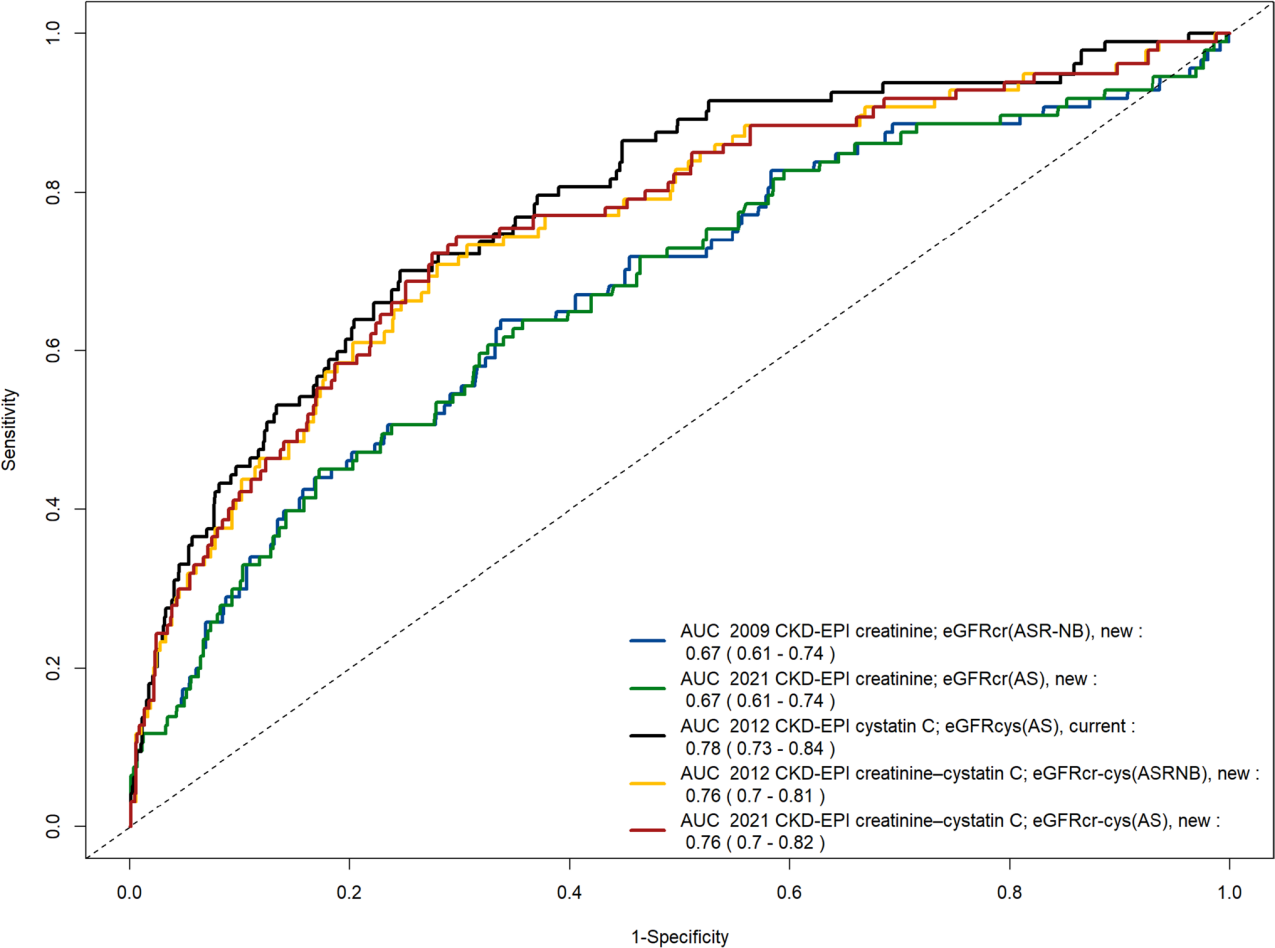
Receiver-operating characteristics curves for cardiovascular mortality after 4 years for all GFR-estimating equations. Additionally, the respective areas under the curve and CI (AUC) are given

**Fig. 3:**
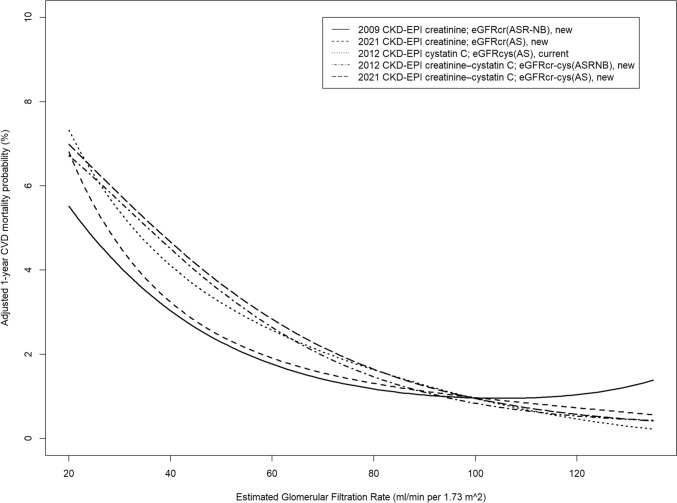
Adjusted 1 year cardiovascular mortality by eGFR. Probabilities were computed for a white woman with the lowest risk category for categorical covariates (smoking status, diabetes status, and BP category) and the overall mean values of continuous covariates (age, body mass index, LDL and HDL cholesterol, log triglycerides, and log CRP)

Receiver-operating characteristics curves for cardiovascular mortality after 4 years for all GFR-estimating equations and the respective areas under the curve are given in Fig. 2. In all Cox regression analyses, the estimated GFR was an independent predictor of cardiovascular death regardless of the equation used, adjusted for age, sex, BMI, diabetes mellitus, current smoking, dyslipidemia, and hypertension (Table 1). As an example, adjusted 1 year cardiovascular mortality probabilities by eGFR estimates were computed for a white woman with the lowest risk category for categorical covariates and the mean values of continuous covariates (Fig. 3).

**Table 1:** Baseline characteristics of patients for the total sample and grouped by 2021 CKD–EPI creatinine–cystatin C; eGFRcr-cys(AS), new

Characteristic	All (*n* = 2089)	eGFR > 120 (*n* = 195)	eGFR 90–120 (*n* = 1183)	eGFR 60–90 (*n* = 572)	eGFR 30–60 (*n* = 130)	eGFR < 30 (*n* = 9)
Age (years)	63.0 (55.0, 69.0)	49.0 (43.5, 54.0)	62.0 (54.0, 67.0)	68.0 (63.0, 72.0)	71.0 (66.2, 75.0)	72.0 (68.0, 75.0)
Men (%)	1622 (77.6)	174 (89.2)	976 (82.5)	393 (68.7)	77 (59.2)	2 (22.2)
Body mass index (kg/m^2^)	27.4 (25.0, 30.1)	26.6 (24.2, 29.3)	27.1 (25.0, 29.9)	27.9 (25.4, 30.6)	27.5 (25.0, 30.9)	29.1 (27.3, 29.4)
Diabetes mellitus (%)	455 (21.8)	19 (9.7)	214 (18.1)	160 (28.0)	58 (44.6)	4 (44.4)
Current smoking (%)	402 (19.2)	70 (35.9)	228 (19.3)	84 (14.7)	19 (14.6)	1 (11.1)
Dyslipidaemia (%)	1526 (73.0)	135 (69.2)	854 (72.2)	432 (75.5)	98 (75.4)	7 (77.8)
Hypertension (%)	1603 (76.7)	115 (59.0)	891 (75.3)	472 (82.5)	116 (89.2)	9 (100)
Creatinine (mg/dL)	0.9 (0.8, 1.1)	0.8 (0.7, 0.9)	0.9 (0.8, 1.0)	1.0 (0.9, 1.2)	1.4 (1.2, 1.6)	2.2 (2.0, 2.5)
Cystatin C (mg/L)	0.8 (0.7, 1.0)	0.6 (0.6, 0.7)	0.8 (0.7, 0.8)	1.0 (0.9, 1.1)	1.5 (1.3, 1.6)	2.2 (1.9, 2.8)

Continuous variables are presented as median values, 25th and 75th percentile. Binary variables are presented as absolute and relative frequencies

The equations which included cystatin C showed higher C-index compared to those which did not include cystatin C (0.75–0.76 vs. 0.71, respectively) (Table 2). The new race-free eGFR equations showed comparable C-indices with the older race-based equation, i.e., 0.718 for the creatinine-based equations and 0.754 for the equation based on creatinine and cystatin C (Table 3).

**Table 2:** Regression of eGFR equations on outcomes

Regression of eGFR equations on cardiovascular death	Hazard ratio	95% confidence interval		*p* value
2009 CKD–EPI creatinine; eGFRcr(ASR-NB), new	0.9751	0.9630	0.9873	< 0.001
2021 CKD–EPI creatinine; eGFRcr(AS), new	0.9756	0.9641	0.9872	< 0.001
2012 CKD–EPI cystatin C; eGFRcys(AS), current	0.9669	0.9582	0.9757	< 0.001
2012 CKD–EPI creatinine–cystatin C; eGFRcr-cys(ASR-NB), new	0.9661	0.9562	0.9761	< 0.001
2021 CKD–EPI creatinine–cystatin C; eGFRcr-cys(AS), new	0.9672	0.9579	0.9767	< 0.001

Provided are hazard ratios and 95% confidence intervals

**Table 3:** C-indices for the association between eGFR equations and cardiovascular death

C-Index for the outcome of cardiovascular death	C-index	95% confidence intervals	
2009 CKD–EPI creatinine; eGFRcr(ASR-NB), new	0.717	0.711	0.723
2021 CKD–EPI creatinine; eGFRcr(AS), new	0.718	0.661	0.775
2012 CKD–EPI cystatin C; eGFRcys(AS), current	0.763	0.708	0.818
2012 CKD–EPI creatinine–cystatin C; eGFRcr-cys(ASR-NB), new	0.753	0.698	0.808
2021 CKD–EPI creatinine–cystatin C; eGFRcr-cys(AS), new	0.754	0.699	0.809

Provided are C-indices and 95% confidence intervals

## Discussion

The present analysis shows that among the new race-free eGFR equations used to estimate eGFR, the equations which include cystatin C are more strongly associated with cardiovascular death compared to race-free equations which include creatinine only, showing a 4–5% increase in the C-statistic. This finding extends the results of previous analyses that showed that cystatin C-based equations were more strongly associated with cardiovascular risk compared to creatinine-based equations which included race [13].

Our finding that the new race-free cystatin C-based equations outperform the equations which do not include cystatin C for the prediction of the risk of cardiovascular death is in line with the previous studies which showed that cystatin-based equations are a better eGFR estimator than creatinine-only equations. In a retrospective individual-level data analysis of > 60,000 participants from five general population and three chronic kidney disease US-based cohorts, it was shown that the eGFRcr-cys equation may be preferred over the race-free, creatinine-only based eGFR equation for assessing racial differences in the risk of kidney failure with replacement therapy and mortality associated with low eGFR [14]. In an analysis by the CKD–EPI collaboration, race-free cystatin C-based equations were more accurate than race-free creatinine-only based equations [5]. In similar, an analysis of the Chronic Renal Insufficiency Cohort study showed that the validity and precision of estimation of GFR from serum cystatin C were similar with those from creatinine, without the need to take into consideration either race or ancestry [15]. In addition, cystatin C-based equations predict eGFR more accurately than creatinine-based equations in children [16], older adults [17], and acutely ill persons [18]. Moreover, early stages of renal function decline are detected more easily by cystatin C-based equations [13]. Additionally, drug dosing and monitoring based on cystatin C result in better achievement of trough levels [19]. For all these reasons, the use of cystatin C-based GFR-estimating equations, alone or combined with creatinine, is strongly recommended to eliminate the use of race in GFR-estimating equations [20].

Cystatin C is a 13-kDa low-molecular-weight protein which is produced in all nucleated human cells at a constant rate, and is freely filtered at the glomerular membrane and metabolized in the proximal renal tubule. The rate of production of cystatin C is not related to muscular mass and hence is not affected by age, sex, or race [19]. Its role as a marker of eGFR was identified in 1979, and it was standardized in 2010 [21]. Since then, cystatin C has been widely used in research, but its role in routine clinical practice is still limited. Reasons for this include the higher associated cost and the slow return of results. It is expected that the costs will decrease when testing increases. The recent recommendations by the National Kidney Foundation and the American Society of Nephrology Task Force might accelerate this [20].

The elimination of race from eGFR equation is only one aspect out of a bundle of many consistent efforts to fight racism, promote justice, diversity, equity, and inclusion, and remove any racial inequities from medical practice and research. Other steps include the identification and adjustment of guidelines, algorithms, educational materials, beliefs, examinations, prognostic tools, and approaches which treat race as a biological rather than a social construct [22]. The removal of racial inequities from health care will improve patient outcomes and promote population health.

The strengths of the study include the large number of patients and the long follow-up, as well as the assessment of a hard outcome like cardiovascular death. A limitation of the study is that the population studied included only individuals of European descent.

In conclusion, this analysis from the Athero*Gene* study shows that the equations for the estimation of eGFR which include cystatin C are more accurate in predicting cardiovascular death compared to the race-free equations which include creatinine only. This finding adds to the related literature which supports the elimination of the use of race in GFR-estimating equations, and promotes the use of cystatin C.

### Supplementary Information

Below is the link to the electronic supplementary material.

Supplementary file1 (DOCX 20 KB)

## Data Availability

Data are available upon reasonable request.
