# Supplementary material for: New race-free creatinine- and cystatin C-based equations for the estimation of glomerular filtration rate and association with cardiovascular mortality in the AtheroGene study
Source: Intern Emerg Med. 2024 Feb 13;19(3):697–703. doi: 10.1007/s11739-023-03529-9 (PMC11039520; doi:10.1007/s11739-023-03529-9)
Supplement: Supplementary file 1 — Supplementary file1 (DOCX 20 KB) [file 11739_2023_3529_MOESM1_ESM.docx]

**Supplemental table** **1:** Constants used in the eGFR equations

| **Model; Name of Equation** | **Intercept μ (95% CI)** | **Coefficients for Creatinine** | | **Coefficients for Cystatin C** | | **Coefficient c for Age** | **Coefficient d for Female Sex** |
| --- | --- | --- | --- | --- | --- | --- | --- |
|  |  | **a1** | **a2** | **b1** | **b2** |  |  |
| 2009 CKD-EPI creatinine; eGFRcr(ASR-NB), new | 141 | -0.329 | -1.209 | NA | NA | 0.9929 | 1.018 |
| 2021 CKD-EPI creatinine; eGFRcr(AS), new | 142 | -0.241 | -1.2 | NA | NA | 0.9938 | 1012 |
| 2012 CKD-EPI cystatin C; eGFRcys(AS), current | 133 | - | - | -0.499 | -1.328 | 0.9962 | 0.932 |
| 2012 CKD-EPI creatinine–cystatin C; eGFRcr-cys(ASR-NB), new | 135 | -0.248 | -0.601 | -0.375 | -0.711 | 0.9952 | 0.969 |
| 2021 CKD-EPI creatinine–cystatin C; eGFRcr-cys(AS), new | 135 | -0.219 | -0.544 | -0.323 | -0.778 | 0.9961 | 0.963 |

Data are derived from [[2](#_ENREF_2)],[[3](#_ENREF_3), [5](#_ENREF_5)]
